# Coronary Sinus Reducer Improves Myocardial Perfusion in a Patient With Angina, Hypertrophic Cardiomyopathy, and Coronary Microvascular Disease

**DOI:** 10.1016/j.cjco.2024.07.011

**Published:** 2024-07-25

**Authors:** Stephen P. Hoole, Katharine Tweed, Lynne Williams, Jonathan Weir-McCall

**Affiliations:** a Royal Papworth Hospital NHS Foundation Trust, Cambridge, United Kingdom; b Department of Cardiovascular Medicine, University of Cambridge, Cambridge, United Kingdom; c Victor Phillip Dahdaleh Heart Lung Research Institute, Cambridge, United Kingdom

**A 69-year-old woman with incapacitating central chest pain despite 3 antianginal medications at maximum tolerated doses and hypertrophic cardiomyopathy with no evidence of left ventricular outflow tract obstruction but extensive anterior subendocardial perfusion abnormality due to coronary microvascular disease was considered for a coronary sinus reducer to treat refractory angina.**

**Learning Objectives**

• To recognise the challenges of treating patients with angina pectoris and HCM caused by CMD.
• To understand the role and mechanism of coronary sinus reducer treatment of CMD in a patient with HCM.

**Novel Teaching Points**

• Coronary sinus reducer (CSR) may be a useful treatment for patients with HCM and angina due to CMD.
• Improvement in diastolic function after CSR is observed as well, consistent with CMD improvement being linked with lusitropy.
• The mechanism appears to be related to augmentation of regional myocardial blood flow in territories with poor blood flow. We also demonstrate improvement in global blood flow.
• Systematic evaluation of the therapeutic potential of CSR in HCM is warranted.

## History of Presentation

We present the case of a 69-year-old woman with nonobstructive hypertrophic cardiomyopathy (HCM) confirmed on genetic testing, echocardiography, and cardiac magnetic resonance (CMR) imaging and a long history of atypical chest pains who then developed recurrent chest pain at rest radiating to her jaw, with breathlessness, nausea, and palpitations associated with small troponin rises. She was diagnosed with a non–ST-segment elevation myocardial infarction complicated by nonsustained ventricular tachycardia. Coronary computed tomographic angiography documented unobstructed coronary arteries, and she was initially treated medically.

## Past Medical History

The patient previously described atypical stabbing chest pains since childhood, and although she was noted to have a murmur, she was a police cadet in her 20s, was able to carry 3 pregnancies to term, and enjoyed an active lifestyle, running several half-marathons into her late 50s. However, in her early 60s, she started to develop frequent exertional chest discomfort that progressed to chest pain at rest. She did not have hypertension.

Echocardiography documented asymmetrical anteroseptal hypertrophy, which was confirmed on CMR with a maximum left ventricular (LV) wall thickness of 17 mm and patchy mesocardial late gadolinium enhancement indicating moderate fibrosis in the hypertrophied segments.

Establishing antianginal therapy with trials of beta-blocker, calcium channel blocker, nitrate, nicorandil, and ranolazine was complicated by hypotension and initially not particularly effective, although ranolazine helped. She was maintained on ranolazine 750 mg twice daily, slow-release diltiazem 90 mg twice daily, and slow-release isosorbide mononitrate 60 mg once daily, but with persistent Canadian Cardiovascular Society (CCS) class 3 angina, a coronary sinus reducer was considered. This medication remained unaltered during her subsequent management.

## Management

**Figure 1:**
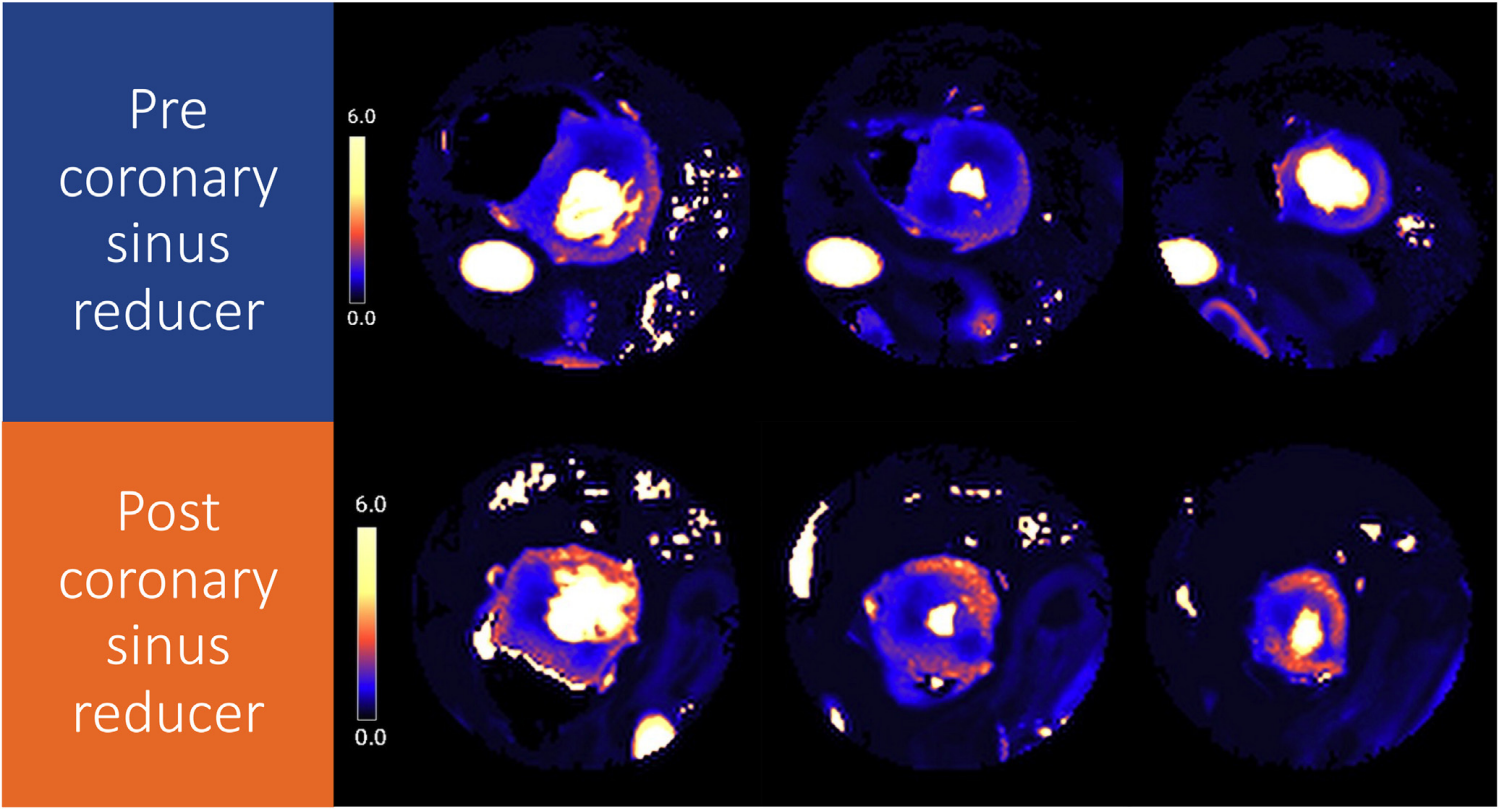
Cardiac magnetic resonance imaging pixel-wise quantitative stress perfusion maps of the basal, mid-, and apical ventricle before and after coronary sinus reducer. On the preprocedural images there is severe global hypoperfusion, most pronounced in the areas of maximal hypertrophy in the basal and mid-anteroseptum and inferior segments. After the procedure there is a significant increase in the global blood flow. Although there is persistent hypoperfusion in the anteroseptum and inferior wall, the extent and severity of this has reduced.

**Figure 2:**
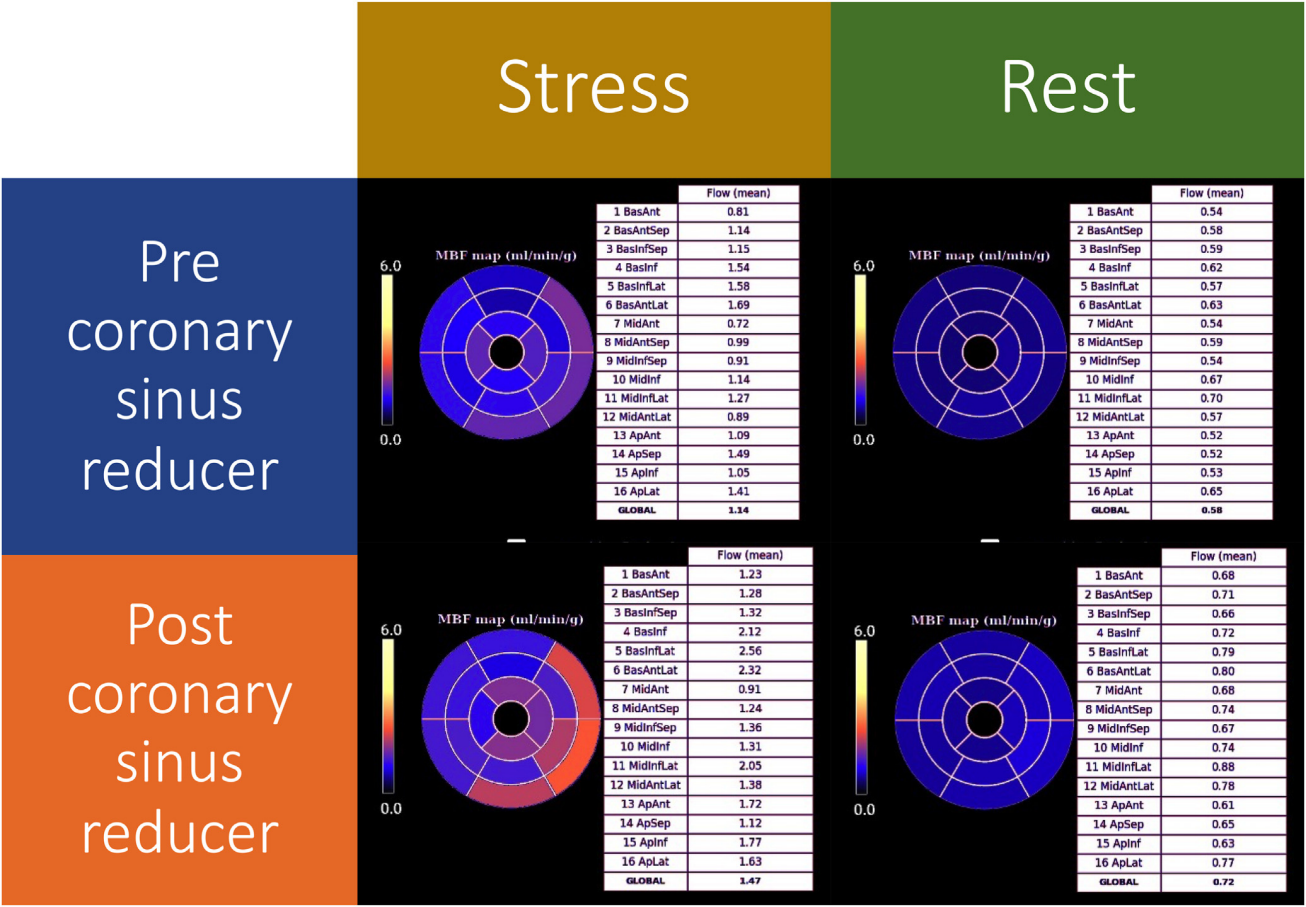
Cardiac magnetic resonance imaging myocardial blood flow maps at stress and rest before and after coronary sinus reducer. On the preprocedural images there is severe global hypoperfusion, most pronounced in the basal anterior wall and mid-septum. After the procedure there is a significant increase in the global blood flow, with the greatest improvements in the regions with the poorest baseline myocardial blood flow.

Preprocedural score on the abbreviated Seattle Angina Questionnaire 7 (SAQ-7) was 38, confirming significant angina burden despite maximal tolerated medical therapy. Echocardiography confirmed grade 2 (pseudo-normalised) diastolic LV dysfunction on mitral inflow velocity and tissue Doppler (mitral valve E/A 1.20, lateral e′ 4.9 cm/s, E/e′ 13.3). 3T quantitative stress perfusion cardiac magnetic resonance (spCMR) confirmed preserved LV ejection fraction of 70%, but a large perfusion defect in the anterior wall and septum extending from the basal to mid-ventricular cavity during adenosine stress (Figs. 1 and 2; Video 1 , view video online). Stress myocardial blood flow (sMBF) was globally reduced (1.14 mL/min/g), as was the myocardial perfusion reserve (MPR; 1.9) with this most pronounced in basal to mid-anterior wall (sMBF 0.8 mL/min/g, MPR 1.5).

Coronary sinus reducer was implanted via the right internal jugular vein and inflated to 5 atmospheres for 1 minute without complication (Supplemental Fig. S1). The patient was prescribed dual antiplatelet therapy with 75 mg clopidogrel and 75 mg aspirin for 6 months. At 6-month follow-up, her angina was less frequent and intense, with a SAQ-7 score that was markedly better at 65 and a CCS class of 2. Follow-up quantitative spCMR indexes, performed under similar conditions, showed improved myocardial perfusion, with global sMBF increasing to 1.5 mL/min/g and MPR increasing to 2.0, with the most pronounced improvement in the basal to mid-anterior wall (sMBF 1.23 mL/min/g, MPR 1.8) (Figs. 1 and 2; Video 2 , view video online). Echocardiographic measures of diastolic dysfunction also confirmed improvement at follow-up (mitral valve E/A 0.85, lateral e′ 5.7 cm/s, E/e′ 7.7).

## Discussion

Patients with HCM are susceptible to CMD. Subendocardial perfusion abnormality causing angina is well recognised during stress as due to a combination of reduced capillary density in LVH and high LV cavity pressure and wall stress.^1^ There is a significant negative association between hyperemic MBF and wall thickness and a higher probability of fibrosis in segments with diminishing MBF during stress. There are few specific therapies for CMD in HCM, but CSR may hold potential.

The CSR (Reducer; Shockwave, a Johnson & Johnson Company) is an hourglass-shaped stainless steel balloon–mounted stent that may be implanted into the coronary sinus to delay myocardial venous outflow. The increased venous back pressure is thought to open and recruit capillary beds, relieving microcirculatory resistance and redistributing blood flow to regions of hypoperfusion, although this has not been definitely proven. A recent sham-controlled randomised trial of CSR for refractory angina confirmed improvement in angina but without improvement of transmural myocardial perfusion as assessed with spCMR, although there was an improvement in endo:epi ratio.^2^ Case-series data in patients with ischemia nonobstructive coronary artery have shown that CSR can improve angina and CMR-measured myocardial perfusion reserve index in ischemic segments and globally.^3^ Other mechanistic studies are on-going (eg, NCT05492110). Blood flow redistribution by capillary recruitment may be particularly attractive in HCM, where subendocardial capillary compression from high LV intracavity pressure and wall stress are thought to be the dominant pathophysiologic mechanism. Through reductions in CMD and ischemia, lusitropy may also be improved, which is also particularly important in noncompliant HCM ventricles.^4^

The present case report, using quantitative spCMR in a patient with HCM, confirms that CSR can improve global MBF and MPR and reduces CMD, with mirrored improvements in angina and diastolic LV dysfunction. The greatest gains were seen in the most hypoperfused segments at baseline. A previous case report of CSR in HCM with the use of positron emission tomography has similarly shown an improvement in quantitative MBF and angina.^5^ The European Society of Cardiology chronic coronary syndromes guidelines have deemed CSR for refractory angina class IIb, level of evidence B, following supporting evidence from case series and a sham-controlled randomised double-blind clinical trial,^6^ with a further randomised trial already underway (NCT05102019). Further systematic evaluation of the therapeutic potential of CSR and particularly studies focused on mechanism of action in patients with evidence of refractory angina and CMD, including those with HCM, are warranted.

## Conclusion

We demonstrate in a case of HCM with angina and CMD that CSR is effective in ameliorating the symptoms of angina by increasing myocardial blood flow to hypoperfused myocardial segments and globally, leading to an improvement in LV diastolic function.

## Supplementary Material

Video 1 Stress perfusion cardiac magnetic resonance imaging before coronary sinus reducer.

Video 2 Stress perfusion cardiac magnetic resonance imaging after coronary sinus reducer.
