# Supplementary material for: Coronary Sinus Reducer Improves Myocardial Perfusion in a Patient With Angina, Hypertrophic Cardiomyopathy, and Coronary Microvascular Disease
Source: CJC Open. 2024 Jul 25;6(11):1299–302. doi: 10.1016/j.cjco.2024.07.011 (PMC11583869; doi:10.1016/j.cjco.2024.07.011)

Supplemental Figure S1

Fluoroscopic images (in LAO +30 degrees) of (A) coronary sinogram demonstrating the coronary sinus anatomy and dimensions, (B) implantation of balloon mounted CSR and (C) immediate post-implantation with balloon markers across the device and (D) post-implantation coronary sinogram demonstrating the device straddling a venous valve as intended.

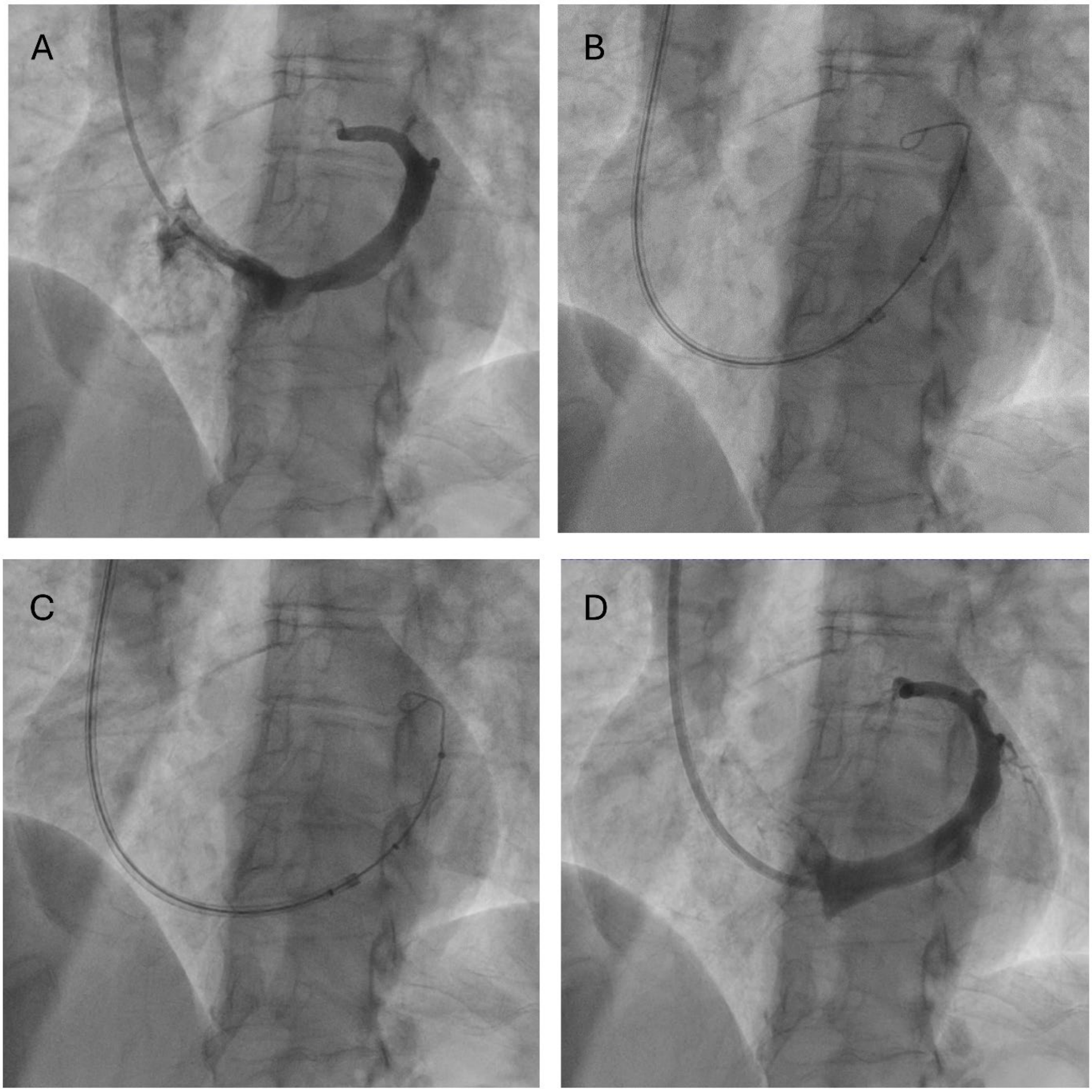

Supplement: Supplementary Figure S1 [file mmc3.pdf]
